# Multi‐center Retrospective Study of Factors Affecting Perioperative Transfusion of Packed Red Blood Cells for Pelvic Fracture Patients

**DOI:** 10.1111/os.13330

**Published:** 2022-07-12

**Authors:** Xiuqiao Xie, Yuanshuai Huang, Xueyuan Huang, Rong Gui

**Affiliations:** ^1^ Department of Blood Transfusion Affiliated Hospital of Southwest Medical University Luzhou China; ^2^ Department of Blood Transfusion Third Xiangya Hospital of the Central South University Changsha China

**Keywords:** Erythrocyte transfusion, Fracture fixation, Fracture, bone classification, Hemoglobins analysis, Pelvic bones injuries, Shock, hemorrhagic

## Abstract

**Objective:**

To analyze the use of packed red blood cells (PRBCs) for patients with pelvic fracture and evaluate factors associated with PRBC transfusion for patients with pelvic fracture.

**Methods:**

This retrospective cohort study collected 551 patients with pelvic fractures from six hospitals between September 1, 2012, and June 31, 2019. The age span of patients varied from 10 to 95 years old, and they were classified into two groups based on high‐energy pelvic fractures (HE‐PFs) or low‐energy pelvic fractures (LE‐PFs). The study's outcome was the use of PRBCs, fresh frozen plasma (FFP), and albumin. Demographic data, characteristics, laboratory tests, clinical treatment details, and clinical outcomes were compared between the two groups. Factors that were statistically associated with perioperative PRBCs in univariate analyses were included to conduct an optimal scale regression to determine the independent factors for perioperative PRBCs.

**Results:**

A total of 551 patients were screened from six hospitals, and after inclusion and exclusion, 319 were finally included and finished the follow‐up from admission to discharge, while four patients died during hospitalization. Three hundred and nineteen patients were classified into two groups by their injury mechanisms. A total of 230/319 (72.1%) patients were classified into the HE‐PF group, and 89/319 (27.8%) patients were classified into the LE‐PF group. Patients in the HE‐PF group were transfused with 4.5 (3–8) units of PRBCs, 300 (0–600) ml of FFP, and 0 (0–30) g of albumin, while patients in the LE‐PF group were transfused with 3.5 (2–4.5) units of PRBCs, 0 (0–295) ml of FFP, and 0 (0–0) g of albumin (all *P* < 0.001). There were higher proportions of male patients and patients under 65 in the HE‐PF group (all *P* < 0.001). HE‐PF group patients were more severely injured and likely to take external fixation. The optimal scale regression revealed four significant factors associated with perioperative transfused PRBCs, which were patients on admission with hemorrhagic shock (importance = 0.283, *P* = 0.004), followed by fracture types identified by Tile classification (importance = 0.156, *P* < 0.001), hemoglobin levels below 70 g/L on admission (importance = 0.283, *P* = 0.004), followed by fracture types identified by Tile classification (importance = 0.156, *P* < 0.001), hemoglobin levels below 70 g/L on admission (importance = 0.148, *P* = 0.039), and methods of pelvic fixation (importance = 0.008, *P* = 0.026), ranked by the importance.

**Conclusion:**

Patients with HE‐PFs had increased transfusions of PRBCs, FFP, and albumin, and hemorrhagic shock on admission, Tile classification, Hb levels, and stabilization methods were found to be associated with perioperative PRBCs.

## Graphical abstract

We discovered that patients with presence of hemorrhagic shock on admission, Tile classification, hemoglobin levels below 70 g/L on admission, and pelvic fixation methods affect the perioperitive transfusion of packed red blood cells (PRBCs).

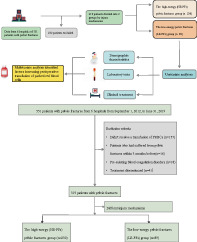

## Introduction

High‐energy pelvic fractures (HE‐PFs) are commonly seen in traffic accidents and falls from heights,^1^ and patients are often severely injured with high risks of mortality and complications.^2^ Low‐energy pelvic fractures (LE‐PFs) are low‐energy trauma mainly observed in the elderly because of osteoporosis and falls on the ground.^3^

Patients with HE‐PFs require a multidisciplinary team approach to address their complex life‐threatening lesions, and the Damage Control Orthopedics protocol is mainly applied.^4^ Patients first receive temporary fracture stabilization to avoid surgery's traumatic effect and will undergo a definitive fracture fixation surgery when suitable for surgery. Red blood cell transfusion is indispensable for patients with HE‐PFs by playing an essential role in saving their lives during their emergency treatment of life‐threatening bleeding, and continuing to perform profoundly in subsequent treatment of the possible hemorrhage induced by surgeries or possible coagulopathy. Patients with HE‐PFs have a higher demand for packed red blood cells (PRBCs).^5^ Less critical than high‐energy fractures and often requiring only conservative treatment, most patients with LE‐PFs need blood transfusion due to chronic anemia and cardiovascular disease history.^6^

Although necessary for orthopedic patients' treatment, the transfusion of allogeneic red blood cells is controversial. In addition to a safe blood supply, people are aware of the unwanted increased mortality and complications related to blood transfusion for patients undergoing surgeries.^7^, ^8^ Amid the controversy, patient blood management (PBM), as a multidisciplinary and multiprofessional approach for better prognoses of patients and the reasonable utilization of blood products, is recommended. Previous studies have confirmed the effectiveness of adequately conducted PBM for orthopedic patients undergoing surgeries on decreasing transfusions and increasing better prognoses.^9^ As the implementation of PBM for orthopedic patients continues through the whole process from admission to discharge, covering every aspect from optimization of hemostasis and correction of anemia to reduction of hemorrhage, it is of significance to identify factors associated with the transfusion of PRBCs to help reinforce the implementation of PBM.

Based on previous studies, the severity of pelvic ring disruption was positively correlated with the overall red blood cell transfusion for orthopedic patients; open book fractures, whether based on fracture classifications of Tile classification or Young–Burgess classification, were associated with more transfusion requirements.^10^, ^11^ PRBCs are necessary for resuscitating injured patients, and the demand for PRBCs is often boosted by trauma‐induced exsanguination.^12^, ^13^ Undetected preexisting bleeding disorders can manifest and even deteriorate for traumatic patients who undergo surgeries.^14^ Additionally, acute traumatic coagulopathy related to the injury is relatively common in unstable pelvic fractures and can increase bleeding tendency.^15^ Medications such as anticoagulant agents are another critical factor in the bleeding tendency of patients undergoing major orthopedic surgery.^16^

Most studies have implied possible factors that could increase the tendency of PRBC transfusion, but they mainly focused on one element of transfusion or the use of PRBCs during a specific period of inpatient time. Since the transfusion of PRBCs’ results from a multitude of factors and the overall transfusion of PRBCs during hospitalization has never been evaluated, our study was designed to: (i) evaluate the perioperative transfusion of PRBCs; and (ii) identify independent predictors for the perioperative transfusion of PRBCs, and help to draw attention to such patients with more effective implementation of PBM on them.

## Methods

### Inclusion and Exclusion Criteria

The inclusion criteria were as follows: (i) patients who received internal fixation (IF), external fixation (EF), or conservative treatment for pelvic stabilization; and (ii) patients who were admitted with diagnoses of pelvic fractures.

The exclusion criteria were as follows: (i) patients who did not receive any transfusion of PRBCs; (ii) patients who had suffered from pelvic fractures 3 months before admission; (iii) patients who had preexisting blood coagulation disorders; and (iv) patients who were transferred from or to another hospital, or abandoned treatment in the course of treatment.

We collected data on the demographics, characteristics, laboratory tests, therapeutic courses, and clinical outcomes of 551 patients from electronic patient systems of six different hospitals between September 1, 2012, and June 31, 2019. HE‐PFs were high‐energy caused injuries, including traffic accidents, falls from height, and industrial and agricultural injuries, while LE‐PFs were low‐energy caused injuries, including falls to the ground and low‐energy external forces.

### Surgical Technique

#### Internal Fixation

##### Anesthesia and Position

All procedures were performed under general anesthesia with the patient in a supine or lateral position as needed.

##### Exposure and Implantation

**Fig. 1:**
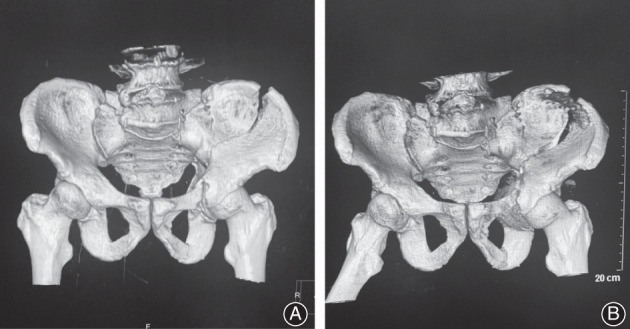
A 63‐year old male patient with HE‐PFs. (A) CT showed bilateral acetabulum, bilateral superior and inferior ramus of pubis, right sacrum, and iliac crest fractures. (B) Another pelvic radiography of the same patient was taken 1 week after open reduction and internal fixation

A 5 cm long incision was made at the anterior superior iliac spine to expose the iliac fracture. Another 15 cm long incision was made along the hip to expose the pelvic fracture end and the posterior wall of the acetabulum through the posterior iliac wing. The reduction was held by pelvic reduction forceps (Orthofix). The steel plate (Orthofix) was fixed to the fracture end of the pelvis and the posterior wall of the acetabulum with an appropriate curvature. The depth of the steel plate was measured after drilling, and proper length screws (Orthofix, Bussolengo, Italy) were placed above and below to fix the plate and ilium (Figure 1).

##### Postoperative Treatment

Patients received ICU nursing, anti‐infection, pain relief, and other symptomatic support treatment with regular wound dressing changes and intensive observation.

#### External Fixation

##### Anesthesia and Position

All procedures were performed under general anesthesia with the patient in a supine or lateral position as needed.

##### Exposure and Implantation

**Fig. 2:**
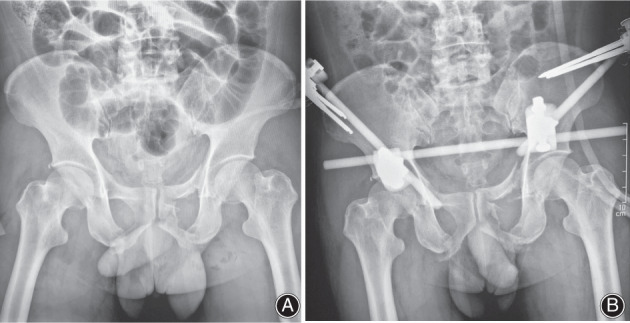
A 50‐year old male patients with HE‐PFs. (A) Pelvic radiograph showed superior and inferior ramus of pubis fracture. (B) Another pelvic radiography of the same patient was taken 1 week after closed reduction and external fixation

Four percutaneous incisions of approximately 1 cm were made along the anterior superior iliac spine and the posterior superior iliac spine; four steel needles (Orthofix) of the external fixator were inserted; after checking the position under the C arm (SSME), the connecting rod (Orthofix) was installed (Figure 2).

##### Postoperative Treatment

Patients received routine orthopedic care, anti‐infection, fluid replacement, and other symptomatic supportive treatment, and they were immobilized in bed, intensively observed, and nursed.

### Conservative Treatment

**Fig. 3:**
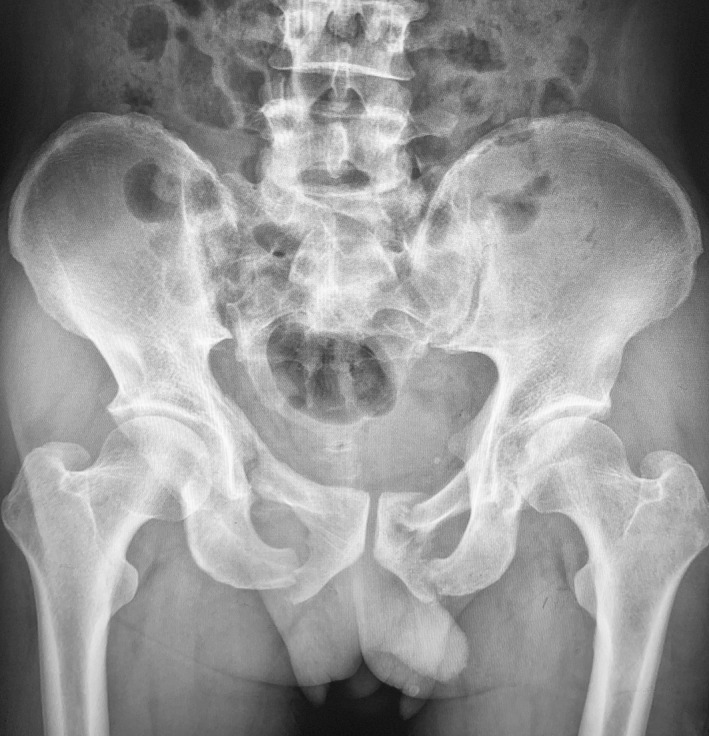
A 57‐year old male patients with LE‐PFs. Pelvic radiograph showed fractures on the left superior and inferior pubic ramus and right inferior pubic ramus

Conservative treatment was administered to patients who did not need surgical treatment, refused surgical treatment, or had contraindications for surgeries. One patient only underwent one pelvic radiograph on the first day after admission, and this radiograph showed a stable pelvic fracture with no indication for surgery (Figure 3).

### Diagnoses and Treatment

Routine blood and conventional coagulation tests on admission were performed to determine the patients' blood flow state and coagulation function. The threshold of 70 g/L was chosen to differentiate hemoglobin (Hb) levels; the cutoff point of hematocrit (Hct) levels was chosen as 30%; the platelet count (PC) of 100 × 10^9^/L was selected as a threshold. The cutoff points of activated partial thromboplastin time (APTT) and prothrombin time (PT) were set to 56 s and 21 s, respectively, 1.5 times those of normal controls. The pelvis Abbreviated Injury Scale represented the severity of pelvic fractures. Types of fractures were identified with Tile classification by a senior orthopedic surgeon based on patient records and radiographs. The American Society of Anesthesiologists (ASA) score was used to classify surgery risk and records of ASA scores were available from patients' anesthesia notes.

Details about the administration of hemostatic drugs, iron supplement agents, the application of autologous blood transfusions, and perioperative transfusions of blood products about PRBCs, FFP, platelets, cryoprecipitate, and albumin were analyzed.

### Outcome Measures

The occurrence of adverse transfusion reactions, including hemolytic transfusion reaction (HTR), allergic transfusion reaction (ATR), and transfusion‐related acute lung injury (TRALI), were recorded. Major complications in orthopedic patients, including deep venous thrombosis (DVT) and pressure ulcers, were recorded too. The Matta radiological grading was used to evaluate the pelvic reduction quality based on the measurement on radiographs of anteroposterior (AP), 40° caudad (inlet), and 40° cephalad (outlet) or Judet views.^17^ The pelvic function was graded by the maximal displacement measured on views as excellent (≤4 mm), good (4–10 mm), fair (10–20 mm), or poor (>20 mm). Death and hospital stay were also recorded.

### Statistical Analysis

All parameters were imported into SPSS® Statistics 25.0 (IBM, Armonk, NY, USA) and analyzed. All descriptive statistics were classified as continuous and categorical variables; continuous variables were tested by the Shapiro–Wilk test to identify their normal distribution. If they were normally distributed, they are presented as the mean ± standard deviation, but if they were not, they are presented as the median with lower and upper interquartile range; categorical variables in the independent variables are presented as numbers (%) or the median with lower and upper interquartile range. The Kruskal—Wallis test or Mann–Whitney U test was used to evaluate differences in nonnormal distributed continuous variables, while differences in categorical variables were assessed by the *χ* ^2^‐test. In univariate analyses of factors associated with perioperative PRBCs, factors with statistical significance and clinical meaning were included to generate an optimal scale model to identify factors associated with the increased perioperative transfusion. A *p* value <0.05 indicated statistical significance.

## Results

### General Results

**Fig. 4:**
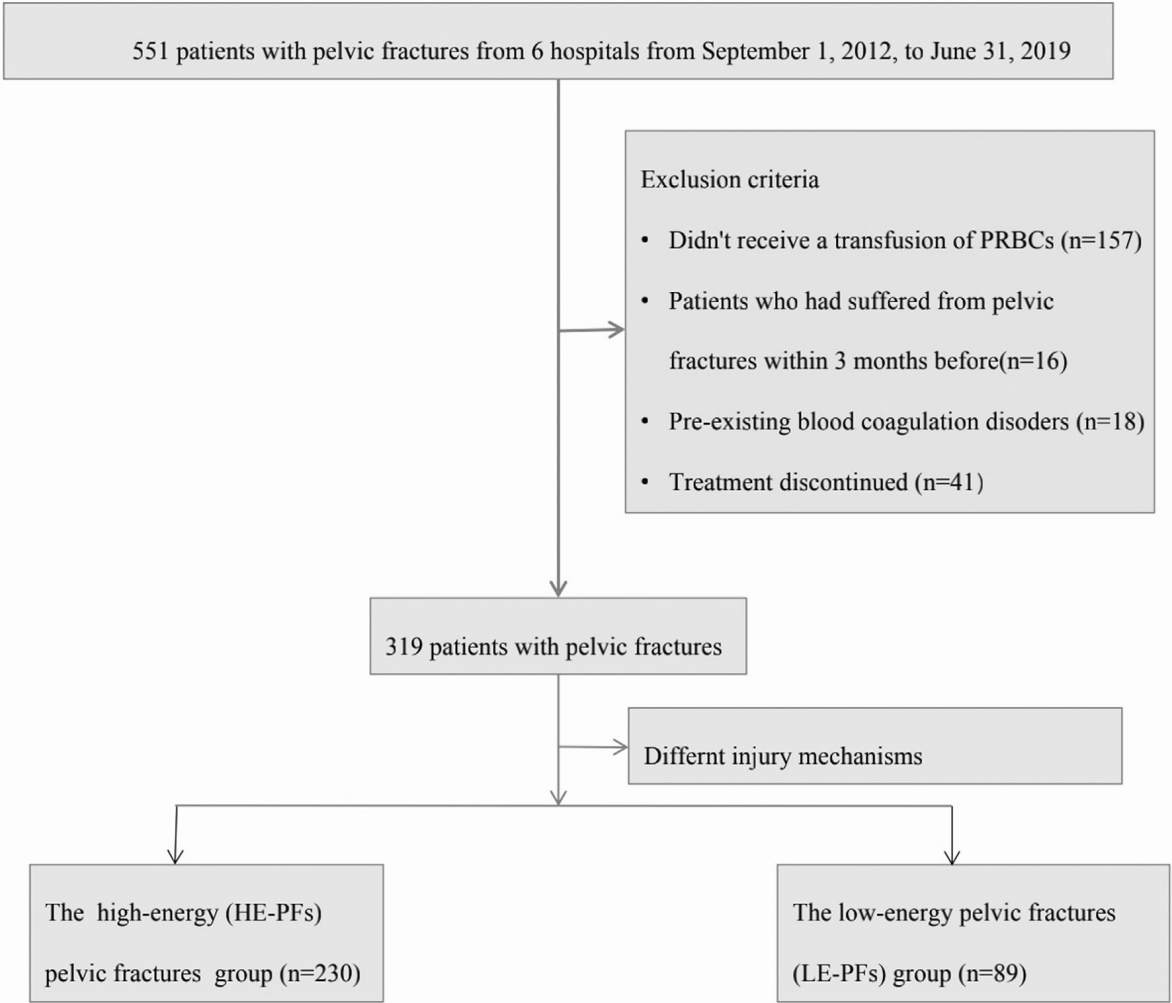
Selection and grouping methods for all 551 patients

A total of 319 patients finished follow‐up of 21 (16–27) days from admission to discharge, and 230 patients were classified into the HE‐PF group, while 89 patients were classified into the LE‐PF group (Figure 4).

There was a higher proportion of male patients in the HE‐PF group (146/230, 63.5%) than in the LE‐PF group (32/89, 36%) (*P* < 0.001). The proportion of patients who were younger than 65 years old was higher in the HE‐PF group (178/230, 77.4%) than in the LE‐PF group (23/89, 25.8%) (*P* < 0.001). The ratios of patients with angiocardiopathy, diabetes, osteoporosis, and other coexisting diseases were higher in the LE‐PF group than in the HE‐PF group (all *P* < 0.01). The ratios of patients with accompanying extremity bone fractures and acetabulum fractures were larger in the HE‐PF group than in the LE‐PF group (all *P* < 0.01). The proportions of patients with associated head injuries, nerve injuries, abdominal injuries, genitourinary injuries, hemorrhagic shock, and other injuries were higher in the HE‐PF group (all *P* < 0.05) (Table 1).

**TABLE 1:** Baseline and characteristics of pelvic fracture patients

	HE‐PF group (*n* = 230)	LE‐PF group (*n* = 89)	Total (*n* = 319)	*χ* ^2^/Fisher's */Z* value	*P* value
Age (years)				73.158	<0.001
<65	178 (77.4%)	23 (25.8%)	201 (63%)		
≥65	52 (22.6%)	66 (74.2%)	118 (37%)		
Gender				19.709	<0.001
Male	146 (63.5%)	32 (36%)	178 (55.8%)		
Female	84 (36.5%)	57 (64%)	141 (44.2%)		
Co‐existing diseases					
Angiocardiopathy	21 (9.1%)	51 (57.3%)	72 (22.6%)	85.209	<0.001
Diabetes	13 (5.7%)	17 (19.1%)	30 (9.4%)	13.623	<0.001
Osteoporosis	2 (0.9%)	50 (56.2%)	52 (16.3%)	143.881	<0.001
Others	20 (8.7%)	18 (20.2%)	38 (11.9%)	8.128	0.004
Accompanying fracture sites					
Skull bones	5 (2.2%)	0 (0%)	5 (1.6%)	/	0.327
Trunk bones	37 (16.1%)	7 (7.9%)	44 (13.8%)	3.648	0.056
Extremity bones	54 (23.5%)	8 (9%)	62 (19.4%)	8.604	0.003
Acetabulum	82 (35.7%)	13 (14.6%)	95 (29.8%)	13.591	<0.001
Associated injuries					
Head injuries	28 (12.2%)	2 (2.2%)	30 (9.4%)	7.422	0.006
Nerve injuries	12 (5.2%)	0 (0%)	12 (3.8%)	/	0.023
Abdominal injuries	98 (42.6%)	2 (2.2%)	100 (31.3%)	48.573	<0.001
Genitourinary injuries	46 (20%)	1 (1.1%)	47 (14.7%)	18.2	<0.001
Retroperitoneal hematoma	8 (3.5%)	0 (0%)	8 (2.5%)	/	0.112
Hemorrhagic shock	47 (20.4%)	3 (3.4%)	50 (15.7%)	14.137	<0.001
Others	94 (40.9%)	11 (12.4%)	105 (32.9%)	23.621	<0.001
Pelvis AIS	3 (2–3)	3 (2–3)	3 (2–3)	−0.046	0.963

Abbreviations: AIS, abbreviated injury scale; HE‐PF, high‐energy pelvic fracture; LE‐PF, low‐energy pelvic fracture.

The percentage of patients discharged with excellent pelvic reduction quality was 17.3% (9/89) in the LE‐PF group and 18.7% (43/230) in the HE‐PF group, with no difference found in the two groups. The percentage of patients discharged with good pelvic reduction quality was 74.2% (66/89) in the LE‐PF group, which was higher than 45.7% (105/230) in the HE‐PF group (*P* < 0.001). The ratio of patients discharged with fair pelvic reduction quality was 23.5% (54/230) in the HF‐PF group, which was higher than 12.4% (11/89) in the LE‐PF group (*P* = 0.027). The ratio of patients discharged with poor pelvic reduction quality was 12.2% (28/230) in the HE‐PF group, which was also higher than 3.4% (3/89) in the HE‐PF group (*P* = 0.017). In the HE‐PF group, two patients died of severe injuries 1 week after admission and one patient died 10 days after admission due to an infection related to an open fracture. One patient in the LE‐PF group with a craniocerebral injury caused by a fall onto the ground died 16 days after admission. The median length of hospital stay was prolonged in the HE‐PF group by 22 (17–29) days, compared with 18 (13–22) days in the LE‐PF group (*P <* 0.001) (Table 2).

**TABLE 2:** Clinical outcomes of pelvic fracture patients

	HE‐PF group (*n* = 230)	LE‐PF group (*n* = 89)	Total (*n* = 319)	*χ* ^2^/Fisher's */Z* value	*P* value
HTR	3 (1.3%)	0 (0%)	3 (0.9%)	/	0.563
ATR	2 (0.9%)	0 (0%)	2 (0.6%)	/	1
TRALI	2 (0.9%)	3 (3.4%)	5 (1.6%)	/	0.135
Pressure ulcers	3 (1.3%)	2 (2.2%)	5 (1.6%)	/	0.621
DVT	6 (2.6%)	0 (0%)	6 (1.9%)	/	0.191
Pelvic reduction quality on discharge					
Excellent	43 (18.7%)	9 (17.3%)	52 (16.3%)	3.465	0.063
Good	105 (45.7%)	66 (74.2%)	171 (53.6%)	20.965	<0.001
Fair	54 (23.5%)	11 (12.4%)	65 (20.4%)	5.89	0.027
Poor	28 (12.2%)	3 (3.4%)	31 (9.7%)	5.668	0.017
Dead	3 (1.3%)	1 (1.1%)	4 (1.3%)	/	1
Hospital stay	22 (17–29)	18 (13–22)	21 (16–27)	−4.395	<0.001

Abbreviations: ATR, allergic transfusion reaction; DVT, deep venous thrombosis; HE‐PF, high‐energy pelvic fracture; HTR, Hemolytic transfusion reaction; LE‐PF, low‐energy pelvic fracture; TRALI, transfusion‐related acute lung injury.

### Diagnoses and Treatment

The ratio of patients diagnosed with Tile A1 fracture was 18% (16/89) in the LE‐PF group, larger than 6.5% (15/230) in the HE‐PF group (*P =* 0.002), and the ratio of patients diagnosed with Tile A2 fracture was 55.1% (49/89) in the LE‐PF group, larger than 27.4% (63/230) in the HE‐PF group (*P* < 0.001). There was a higher proportion of patients diagnosed with Tile B1 fractures in the HE‐PF group (53/230, 23%) than in the LE‐PF group (11/89, 12.4%) (*P* = 0.033), and there were larger proportions of patients diagnosed with Tile C1 and C2 fractures in the HE‐PF group than in the LE‐PF group (all *P* < 0.05) (Table 3).

**TABLE 3:** Tile classification of pelvic fracture patients

	HE‐PF group (*n* = 230)	LE‐PF group (*n* = 89)	Total (*n* = 319)	*χ* ^2^/Fisher's value	*P* value
A1	15 (6.5%)	16 (18%)	31 (9.7%)	9.599	0.002
A2	63 (27.4%)	49 (55.1%)	112 (35.1%)	21.556	< 0.001
A3	9 (3.9%)	0 (0%)	9 (2.8%)	/	0.067
B1	53 (23%)	11 (12.4%)	64 (20.1%)	4.567	0.033
B2	16 (7%)	5 (5.6%)	21 (6.6%)	0.187	0.665
B3	26 (11.3%)	4 (4.5%)	30 (9.4%)	3.493	0.062
C1	16 (7%)	1 (1.1%)	17 (5.3%)	/	0.048
C2	15 (6.5%)	1 (1.1%)	16 (5%)	/	0.049
C3	17 (7.4%)	2 (2.2%)	19 (6%)	3.031	0.082

Abbreviations: HE‐PF, high‐energy pelvic fracture; LE‐PF, low‐energy pelvic fracture.

The results of blood tests on admission showed that 47/319 (20.1%) patients were found to have Hb levels below 70 g/L, 40/230 (21.3%) of whom were in the HE‐PF group and 7/89 (16.9%) of whom were in the LE‐PF group (*P* = 0.031). Patients with Hct levels below 30% were 180/319 (56.4%) in total and 142/230 (61.7%) patients were in the HE‐PF group, while 38/89 (42.7%) patients were in the LE‐PF group (*P =* 0.002) (Table 4).

**TABLE 4:** Results of blood tests on admission associated with transfusion

	HE‐PF group (*n* = 230)	LE‐PF group (*n* = 89)	Total (*n* = 319)	*χ* ^2^/Fisher's value	*P value*
Blood routine tests					
Hb (g/L)				4.635	0.031
<70	40 (21.3%)	7 (16.9%)	47 (20.1%)		
≥70	190 (78.7%)	82 (83.1%)	272 (79.9%)		
Hct (%)				9.464	0.002
<30	142 (61.7%)	38(42.7%)	180(56.4%)		
≥30	88 (38.3%)	51 (57.3%)	139 (43.6%)		
PC (X10^9^/L)				0.792	0.373
<100	49 (21.3%)	15 (16.9%)	64 (20.1%)		
≥100	181 (78.7%)	74 (83.1%)	255 (79.9%)		
PT (s)				/	0.57
<21	217 (94.3%)	86 (96.6%)	303 (95%)		
≥21	13 (5.7%)	3 (3.4%)	16 (5%)		
APTT (s)				1.473	0.225
<56	214 (93%)	86 (96.6%)	300 (94%)		
≥56	16 (7%)	3 (3.4%)	19 (6%)		

Abbreviations: APTT, activated partial thromboplastin time; Hb, hemoglobin; Hematocrit, Hct; HE‐PF, high‐energy pelvic fracture; LE‐PF, low‐energy pelvic fracture; PC, platelet count; PT, prothrombin time.

For emergency surgeries, 14 exploratory laparotomy surgeries were performed, all in the HE‐PF group (*P =* 0.013). More patients in the HE‐PF group chose IF as pelvic stabilization, while more patients in the LE‐PF group chose EF as pelvic stabilization (all *P* *<* 0.001). No differences were found in other elective surgeries performed for patients between the two groups (Table 5).

**TABLE 5:** Types of the in‐hospital procedures performed for pelvic fracture patients

	HE‐PF group (*n* = 230)	LE‐PF group (*n* = 89)	Total (*n* = 319)	*χ* ^2^/Fisher's */Z* value	*P* value
ASA	2 (1–3)	2 (2–2)	2 (1–3)	−0.245	0.807
Emergency surgeries					
Exploratory laparotomy	14 (6.1%)	0 (0%)	14 (4.4%)	/	0.013
Pelvic packing	2 (0.9%)	0 (0%)	2 (0.6%)	/	1
Angiographic embolization	5 (2.2%)	0 (0%)	5 (1.6%)	/	0.327
Ruptured organ resection	7 (3%)	0 (0%)	7 (2.2%)	/	0.197
Stabilization methods					
IF	168 (73%)	36 (40.4%)	204 (63.9%)	29.57	< 0.001
EF	35 (15.2%)	47 (52.8%)	82 (25.7%)	47.482	< 0.001
Conservative treatment	27 (11.7%)	6 (6.7%)	33 (10.3%)	1.728	0.189
Other elective surgeries					
Craniotomy	8 (3.5%)	1 (1.1%)	9 (2.8%)	/	0.453
Extremity fracture fixation	103 (44.8%)	41 (46.1%)	144 (45.1%)	0.043	0.836
Trunk bone fixation	20 (8.7%)	4 (4.5%)	24 (7.5%)	1.628	0.202
Vascular surgeries	14 (6.1%)	4 (4.5%)	18 (5.6%)	0.306	0.58
Others	13 (5.7%)	1 (1.1%)	14 (4.4%)	/	0.123

Abbreviations: ASA, The American Society of Anesthesiologists; EF, external fixation; HE‐PF, high‐energy pelvic fracture; IF, internal fixation; LE‐PF, low‐energy pelvic fracture.

The proportion of patients administered hemostatics was higher in the HE‐PF group (33/230, 14.3%) than in the LE‐PF group (28/89, 3.1%) (*P* < 0.001), and ratio of patients administered iron supplement agents was also higher in the HE‐PF group (83/230, 36.1%) than in the LE‐PF group (5/89, 0.6%) (*P* < 0.001). PRBCs, FFP, and albumin were more frequently transfused in the HE‐PF group (all *P* *<* 0.001); patients in the HE‐PF group were transfused with 4.5 (3–8) units of PRBCs, 300 (0–600) ml of FFP, and 0 (0–30) g of albumin, while patients in the LE‐PF group were transfused with 3.5 (2–4.5) units of PRBCs, 0 (0–295) ml of FFP, and 0 (0–0) g of albumin (Table 6).

**TABLE 6:** Medications and blood products used for pelvic fracture patients

	HE‐PF group (*n* = 230)	LE‐PF group (*n* = 89)	Total	*χ* ^2^/Fisher's */Z* value	*P* value
			(*n* = 319)		
Hemostatic drugs	33 (14.3%)	28 (3.1%)	61 (19.1%)	12.151	<0.001
Iron supplement agents	83 (36.1%)	5 (0.6%)	88 (27.6%)	29.822	<0.001
Autologous blood transfusions	14 (6.1%)	2 (0.2%)	16 (5%)	/	0.252
PRBCs (unit)	4.5 (3–8)	3.5 (2–4.5)	4 (3–7)	−3.766	<0.001
FFP (ml)	300 (0–600)	0 (0–295)	200 (0–600)	−3.821	<0.001
Platelets (unit**)**	0 (0–0)	0 (0–0)	0 (0–0)	−1.249	0.212
Cryoprecipitate (unit)	0 (0–0)	0 (0–0)	0 (0–0)	−1.417	0.157
Albumin (g)	0 (0–30)	0 (0–0)	0 (0–10)	−5.944	<0.001

Abbreviations: FFP, fresh frozen plasma; HE‐PF, high‐energy pelvic fracture; LE‐PF, low‐energy pelvic fracture; PRBCs, packed red blood cells.

### Factors Associated with Perioperative PRBCs

On analyses of factors that were statistically associated with perioperative PRBCs, patients aged <65 years were transfused with more PRBCs than patients ≥65 years (*P =* 0.002), and male patients were transfused with more PRBCs than female patients (*P =* 0.001). Moreover, patients with comorbidities of angiocardiopathy, diabetes, or osteoporosis were transfused with fewer PRBCs than those without such comorbidities, respectively (all *P*s < 0.05).

Patients with associated abdominal injuries, genitourinary injuries, or hemorrhagic shock were transfused with more PRBCs than those without such associated injuries, respectively (all *P*s < 0.01). Patients identified with Tile A1 to C3, were transfused with 4 (2.5–5.5), 3.8 (2–5.5), 7 (1.8–8.5), 4 (3–5.5), 4 (3–8), 4.3 (2.8–8.3), 5.5 (3–11), 6 (3.3–9), and 8.5 (6–10.5) units of PRBCs, respectively (*P* = 0.001). Further analyses of differences between groups showed that there were differences in transfused PRBCs between patients with Tile A2 and C3 (*Z* = −98.311, *P* = 0.001), patients with Tile A1 and C3 (*Z* = −93.423, *P* = 0.017), and patients with Tile B1 and C3 (*Z* = −88.67, *P* = 0.008).

Patients with Hb levels lower than 70 g/L, Hct levels lower than 30%, PCs lower than 100X 10^9^/L, APTTs longer than 56 s, or PTs longer than 21 s, received more PRBCs, respectively (all *P*s < 0.05). Patients who underwent emergency surgeries of exploratory laparotomy, angiographic embolization, or ruptured organ resection received more PRBCs than those who did not undergo such emergency surgeries, respectively (all *P*s *<* 0.05). Methods of pelvic stabilization were associated with different PRBCs; patients who had IF fixation were transfused with 4 (3–7.9) units of PRBCs, patients who had EF fixation with 3.5 (2–5.6) units of PRBCs, and patients who received conservative treatment with 4 (2–6.5) units of PRBCs (*P* = 0.045). Further analysis identified a difference of PRBC transfusion between IF and EF fixation (*Z* = 28.841, *P* = 0.049). Patients who underwent extremity fracture fixation surgeries received more PRBCs than those who did not (*P* = 0.005). Patients administered hemostatic drugs received more PRBCs than those who were not (*P <* 0.001) (Table 7).

**TABLE 7:** Factors that were statistically associated with PRBCs

	No. of patients	PRBCs (unit)	*Z/H* value	*p* value
Age			−3.103	0.002
<65	201 (60.5%)	4.5 (3–8)		
≥65	118 (35.5%)	3.5 (2–5.5)		
Gender			−3.248	0.001
Male	178 (53.6%)	4.5 (3–8)		
Female	141 (42.4%)	3.5 (2–6)		
Angiocardiopathy			−2.24	0.025
Yes	72 (22.6%)	3.5 (2.5–5.9)		
No	247 (77.4%)	4 (3–8)		
Diabetes			−2.086	0.037
Yes	30 (9.4%)	3.3 (2.4–4)		
No	289 (90.6%)	4 (3–7.5)		
Osteoporosis			−4.508	< 0.001
Yes	52 (16.3%)	3 (2–4)		
No	267 (83.7%)	4 (3–8)		
Abdominal injuries			−2.984	0.003
Yes	100 (31.3%)	5.3 (3–8.5)		
No	219 (68.7%)	4 (3–6)		
Genitourinary injuries			−3.157	0.002
Yes	47 (14.7%)	5.5 (4–8.5)		
No	272 (85.3%)	4 (2.6–6.5)		
Hemorrhagic shock			−4.986	< 0.001
Yes	50 (15%)	7.8 (4–17)		
No	269 (85%)	4 (2.4–6)		
Tile classification			25.314	0.001
A1	31 (9.3%)	4 (2.5–5.5)		
A2	112 (33.7%)	3.8 (2–5.5)		
A3	9 (2.7%)	7 (1.8–8.5)		
B1	64 (19.2%)	4 (3–5.5)		
B2	21 (6.3%)	4 (3–8)		
B3	30 (9%)	4.3 (2.8–8.3)		
C1	17 (5.1%)	5.5 (3–11)		
C2	16 (4.8%)	6 (3.3–9)		
C3	19 (5.7%)	8.5 (6–10.5)		
Hb (g/L)			−5.509	< 0.001
<70	47 (14.1%)	8 (4.5–13)		
≥70	272 (81.9%)	4 (2–6)		
Hct (%)			−2.895	0.004
<30	180 (54.2%)	4.5 (3–8)		
≥30	139 (41.8%)	3.5 (2–6)		
PC (X10^9^/L)			−2.869	0.004
<100	64 (19.2%)	5.8 (3–11.8)		
≥100	255 (76.8%)	4 (3–6)		
PT (s)			−2.4	0.016
<21	303 (91.2%)	4 (3–6.5)		
≥21	16 (4.8%)	8 (4.1–14)		
APTT (s)			−3.352	0.001
<56	300 (90.3%)	4 (3–6.5)		
≥56	19 (5.7%)	9 (4–15)		
Exploratory laparotomy			−3.449	0.001
Yes	14 (4.4%)	7.8 (4.9–15.1)		
No	305 (95.6%)	4 (3–6.6)		
Angiographic embolization			−2.302	0.021
Yes	5 (1.6%)	8 (5.5–23.5)		
No	314 (98.4%)	4 (3–7)		
Ruptured organ resection			−3.329	0.001
Yes	7 (2.2%)	13 (6.5–16.5)		
No	312 (97.8%)	4 (3–6.8)		
Stabilization methods			6.187	0.045
IF	204 (63.9%)	4 (3–7.9)		
EF	82 (25.7%)	3.5 (2–5.6)		
Conservative treatment	33 (10.3%)	4 (2–6.5)		
Extremity fracture fixation			−2.813	0.005
Yes	144 (45.1%)	4.5 (3–8)		
No	175 (54.9%)	4 (2–6)		
Hemostatic drugs			−3.585	< 0.001
Yes	88 (26.5%)	4.5 (4–8.5)		
No	231 (69.5%)	4 (2–6)		

Abbreviations: APTT, activated partial thromboplastin time; EF, external fixation; Hb, hemoglobin; Hematocrit, Hct; IF, internal fixation; PC, platelet count; PRBCs, packed red blood cells; PT, prothrombin time.

### Optimal Scale Regression for Perioperative Transfusion of PRBCs

The optimal scale regression model that we conducted was statistically significant (*F* = 4.02, *P* < 0.001, and adjusted *R* ^2^ = 0.295), ensuring that there were statistically significant relations between the included variables and perioperative PRBCs. The correlation and tolerance examination excluded collinear relationships between the independent variables because the tolerance of each variable after conversion was above 0.6. Independent variables with statistical significance were the presence of hemorrhagic shock on admission (importance = 0.283, *P* = 0.004), pelvic fractures identified by Tile classification (importance = 0.156, *P* < 0.001), hemoglobin levels below 70 g/L on admission (importance = 0.148, *P* = 0.039), and methods of pelvic fracture fixation (importance = 0.008, *P* = 0.026) (Table 8).

**TABLE 8:** Optimal scale regression for perioperative transfusion of PRBCs

Independent variable with significance	Assignment	*B* value	*F* value	*P* value	Importance
Hemorrhagic shock on admission	1 = no 2 = yes	0.235	8.495	0.004	0.283
Tile classification	1 = A1 2 = A2 3 = A3 4 = B1 5 = B2 6 = B3 7 = C1 8 = C2 9 = C3	−0.158	10.017	0	0.156
Hb levels	1 = below 70g/L 2 = at and above 70g/L	−0.14	4.313	0.039	0.148
Stabilization methods	1 = IF 2 = EF 3 = Conservative treatment	−0.096	3.699	0.026	0.008

Abbreviations: EF, external fixation; Hb, hemoglobin; IF, internal fixation.

### Complications

There were 3/230 (1.3%) patients in the HE‐PF group who suffered from delayed HTR. One patient was given M antigen‐negative PRBCs in subsequent transfusions after he received 12 units of PRBCs with Hb level decreased and total bilirubin level increased. Transfusion was stopped for the other two patients after they manifested unexplained fever, soy‐sauce‐colored urine, and yellow sclera with detection of anti‐E antibodies on antibody screening for HTR. All patients were relieved FINALLY.

ATR appeared in 2/230 (0.9%) patients in the HE‐PF group. The transfusion of FFP was immediately stopped with decadron and adrenalin given to ONE patient when double eyelids dropsy and urticaria in her upper extremities manifested. Transfusion was timely interrupted with decadron, hydrocortisone, and adrenalin administered to another patient when red papules developed in his forebreast and his blood pressure persistently dropped during PRBC transfusion. Their symptoms were finally relieved.

Patients were diagnosed with TRAIL when acute respiratory distress or acute pulmonary edema symptoms developed within 6 h after blood transfusion. TRALI occurred in 5/319 (1.6%) patients in total, 2/230 (0.9%) patients were in the HE‐PF group, and 3/89 (3.4%) patients were in the LE‐PF group (*P* = 0.135). Blood transfusion was stopped immediately, and they were given symptomatic and supportive treatments with conditions finally in remission.

There were 5/319 (1.6%) patients diagnosed with pressure ulcers during the process of hospitalization, 3/230 (1.3%) of whom were in the HE‐PF group and 2/89 (2.2%) of whom were in the LE‐PF group (*P* = 0.621). Their pressure ulcers were treated with cleanout or debridement, and they were prescribed with antibiotics. Their symptoms were alleviated before discharge. DVT occurred in 6/319 (1.9%) patients, all of whom were all in the HE‐PF group. All six patients were treated with thrombolytic therapy and cured without DVT before discharge (Table 2).

## Discussion

### Patient Characteristics

Previous studies have found several factors connected with red blood transfusion and the impact of red blood transfusion on pelvic fracture patients,^7^, ^8^, ^9^, ^10^ but the inpatient use of red blood cells has never been evaluated. We retrieved data of orthopedic patients from six hospitals, analyzed their perioperative transfusion of red blood cells, and investigated the factors that influenced red cell transfusion for pelvic fracture patients to improve PBM practice in pelvic fracture patients. We excluded patients who previously had coagulation dysfunction, lest their conditions may manifest and deteriorate under trauma and become a confounding factor for increased red cell transfusion. We also excluded patients who had pelvic fractures in the 3 months before admission because they returned to hospitals, mainly because of failed pelvis fixation rather than initial trauma to the pelvis, and they received conservative treatment rather than a second operation. As we analyzed the overall in‐hospital process of pelvic fracture patients who received red blood cell transfusion, we excluded discontinuously treated patients who were transferred from or to another hospital or abandoned treatment.

### The Severity of Injury

High‐energy mechanisms in blunt trauma, such as traffic accidents and falls from height are two leading causes of HE‐PFs.^1^, ^2^ Compared with LE‐PFs, HE‐PFs occur predominantly in men; patients with HE‐PFs are younger and have a higher Injury Severity Score, while patients with low‐energy pelvic fractures are the opposite.^5^ In our univariate analyses for factors associated with perioperative PRBCs, the injury mechanism determined the traumatic situation of pelvic patients, and the demand for PRBCs increased in the HE‐PF patients, as HE‐PFs occurred more frequently in younger, male, and more severely traumatized patients.

HE‐PF patients were more likely to be diagnosed with partially unstable B1 fractures and unstable C1and C2 fractures, while LE‐PF patients were more likely to be diagnosed with stable Tile A1 and A2 fractures. The Tile classification used to classify different magnitudes of pelvic fractures in our study is based on the injury mechanism and the pelvic ring's stability. Compared with stable Tile A fractures, Tile C fractures are known for a bleeding tendency and increased blood transfusion because of their unstable properties.^10^ Our study identified Tile classification as a factor associated with transfusion of PRBCs in the optimal scale regression.

### Hemorrhage on admission

It has been shown that massive hemorrhage can even deteriorate under the stress of retroperitoneal hematoma and hemorrhagic shock.^18^ This accounts for a minority of pelvic fracture patients and necessitates a multidisciplinary approach for emergent control of bleeding,^19^ including a huge demand for red blood cell transfusion. The presence of hemorrhagic shock ranked as the first significant factor positively associated with increased PRBCs in our optimal scale regression.

Blood loss from trauma in pelvic fracture patients can lead to observed hemostatic abnormalities.^20^ Early coagulation monitoring by a traditional laboratory determination of APTT, PT, and PC is strongly recommended. Plasma‐based coagulation strategy plasma, such as FFP, should be administered to maintain PT and APTT <1.5 times normal controls.^21^ Early platelet dysfunction is involved in trauma‐induced coagulopathy, and a higher threshold of 100 × 10^9^/L is suggested for PC in major trauma patients with significant bleeding.^22^ It is found that initial Hct level is closely correlated with blood loss, and an Hct level higher than 30% is required to sustain hemostasis.^23^ Hb, rather than Hct level, is currently widely used as a part of the basic diagnostic workup for trauma patients. While there is no prospective randomized controlled trial available for restrictive and liberal transfusion regimens in a trauma patient, a retrospective study showed that an Hb transfusion trigger of <70 g/L resulted in fewer transfusions than an Hb transfusion trigger of <100 g/L, and an Hb transfusion trigger <70 g/L appeared to be safe in a subset of 203 trauma patients.^24^ Hb levels below 70 g/L on admission were one significant factor positively associated with increased PRBCs in our optimal scale regression.

### Stabilization Methods

Elective pelvic fixation surgeries are performed to recreate the pelvic ring's stability when the patient's trauma is controlled suitable for surgery. IF and EF can provide the best fixation stability and satisfactory effectiveness to stabilize unstable pelvic fractures.^25^, ^26^ Older pelvic fracture patients mostly have stable pelvic fractures, and they rarely require operative treatment, so conservative treatment is preferred.^27^ In addition, those who refuse surgical treatment or contraindicate surgeries would also receive conservative treatment. As the mechanism of injury determines the type of fracture and guides the choice of fixation, more patients in the HE‐PF group chose IF while more patients in the LE‐PF group chose EF in our study. We identified stabilization method as a factor affecting the transfusion of PRBCs.

### Strengths and Limitations

The retrieval of data from six hospitals increased the external validity, but the information bias of retrospectively obtained data was inevitable. We only considered the intervention taken from admission to discharge, while the prehospital care record was unavailable. Further study that considers prehospital care would be needed because prehospital fluid administration might impact the blood volume of patients.^28^ We assumed the patients to be normovolemic without considering weight and height, which was never validated but widely accepted in other studies, but a 5‐year retrospective study found that morbid obesity represented a significant risk factor for posttraumatic transfusion in isolated pelvic trauma.^29^ In another study, body mass index was verified to play a role in adverse transfusion reactions.^30^ Antiplatelet or anticoagulant therapies are observed mainly in geriatric patients, and such medicines may be a confounding factor because patients in the LE‐PF group were primarily geriatric patients in our study. However, we excluded patients who had preexisting blood coagulation disorders; therefore, their influence on the perioperative transfusion of PRBCs would be slight.

### Conclusion

This study identified that patients with HE‐PFs had more severe pelvic fractures and combined injuries, and they demanded more perioperative transfusions of PRBCs, FFP, and albumin than patients with LE‐PFs. The optimal scale regression identified four independent factors positively associated with more perioperative PRBC transfusion: the presence of hemorrhagic shock on admission, Tile classification, methods of pelvic stabilization, and Hb levels on admission.
